# Trouble at the Grady Hospital

**Published:** 1905-04

**Authors:** 


### TROUBLE AT THE GRADY HOSPITAL.

THE unfortunate muddle in Grady Hospital affairs has been adjusted and once more "grim-visaged war has smoothed his wrinkled front" and peace prevails over this temple of Hygeia. The trouble, briefly stated, arose over the alleged tyranny of the head nurse and her methods of enforcing discipline. These methods produced such dissatisfaction and irritation in the corps of nurses under her that they resigned in a body and abandoned their posts of duty. The house-surgeon, apothecary and one of the junior assistants espoused the cause of the nurses, and the apothecary ventilated his views on the subject in a card to the newspapers, which contained certain references to the medical staff of the hospital which were regarded as highly offensive. On being pressed, the apothecary disavowed the authorship of the objectionable card and alleged in defense of himself that it had been inspired by the house-surgeon, whereupon the house-surgeon acknowledged the fact, assumed responsibility for the communication and at the same time tendered his resignation. The junior assistant sympathetically resigned. The nurses, having presented their side of the controversy, became repentant and begged to be reinstated. This request, after much discussion, was finally acceded to with certain punitive conditions. The resignations of the house-surgeon and the assistants, however, were not accepted, but both were dismissed from the service of the hospital. The head-nurse, having vacated her position, some one else more generally acceptable was placed in charge and the episode, that had become a newspaper sensation, was closed.

It is truly regretable that such occurrences arise in the management of the hospital. They afford brilliant opportunities for the newspapers to make sensational stories and to "roast" the institution, arousing in the public mind distrust and suspicion against it.

The public seems to lose sight of the fact that the medical profession gives its best time and efforts unstintingly to the alleviation of the ills of the pauper class. Yet, instead of expressing gratitude, the devoted public not only looks a gift horse in the mouth, but tries to dislocate its jaw in doing so.

